# Exploring the Paradoxical Relationship of a Creb 3 Regulatory Factor Missense Variant With Body Mass Index and Diabetes Among Samoans: Protocol for the Soifua Manuia (Good Health) Observational Cohort Study

**DOI:** 10.2196/17329

**Published:** 2020-07-23

**Authors:** Nicola L Hawley, Alysa Pomer, Anna C. Rivara, Samantha L Rosenthal, Rachel L Duckham, Jenna C Carlson, Take Naseri, Muagututia Sefuiva Reupena, Melania Selu, Vaimoana Lupematisila, Folla Unasa, Lupesina Vesi, Tracy Fatu, Seipepa Unasa, Kima Faasalele-Savusa, Abigail I Wetzel, Christina Soti-Ulberg, Angela T Prescott, Gloria Siufaga, Corina Penaia, Sophie B. To, Lauren C LaMonica, Viali Lameko, Courtney C Choy, Scott E Crouter, Susan Redline, Ranjan Deka, Erin E Kershaw, Zsolt Urban, Ryan L. Minster, Daniel E. Weeks, Stephen T McGarvey

**Affiliations:** 1 Department of Chronic Disease Epidemiology Yale School of Public Health New Haven, CT United States; 2 Department of Human Genetics Graduate School of Public Health University of Pittsburgh Pittsburgh, PA United States; 3 Institute of Physical Activity and Nutrition Deakin University Geelong Australia; 4 Australian Institute for Musculoskeletal Sciences The University of Melbourne and Western Health St Albans Australia; 5 Department of Biostatistics Graduate School of Public Health University of Pittsburgh Pittsburgh, PA United States; 6 Ministry of Health Apia Samoa; 7 Lutia I Puava Ae Mapu I Fagalele Apia Samoa; 8 Obesity, Lifestyle and Genetic Adaptations Study Group Apia Samoa; 9 International Health Institute Department of Epidemiology, School of Public Health Brown University Providence, RI United States; 10 Department of Plastic and Reconstructive Surgery University of Pittsburgh Medical Center Pittsburgh, PA United States; 11 Asian Pacific Islander Forward Movement Los Angeles, CA United States; 12 Department of Social and Behavioral Sciences Yale School of Public Health New Haven, CT United States; 13 Oceania University of Medicine Apia Samoa; 14 Department of Kinesiology, Recreation, and Sport Studies The University of Tennessee Knoxville Knoxville, TN United States; 15 Departments of Medicine Brigham and Women's Hospital and Beth Israel Deaconess Medical Center Harvard Medical School Boston, MA United States; 16 Department of Environmental Health College of Medicine University of Cincinnati Cincinnati, OH United States; 17 Division of Endocrinology Department of Medicine University of Pittsburgh Pittsburgh, PA United States; 18 Department of Anthropology Brown University Providence, RI United States

**Keywords:** cohort studies, CREBRF, type 2 diabetes, obesity, Samoa

## Abstract

**Background:**

The prevalence of obesity and diabetes in Samoa, like many other Pacific Island nations, has reached epidemic proportions. Although the etiology of these conditions can be largely attributed to the rapidly changing economic and nutritional environment, a recently identified genetic variant, rs373863828 (CREB 3 regulatory factor, CREBRF: c.1370G>A p.[R457Q]) is associated with increased odds of obesity, but paradoxically, decreased odds of diabetes.

**Objective:**

The overarching goal of the Soifua Manuia (Good Health) study was to precisely characterize the association of the *CREBRF* variant with metabolic (body composition and glucose homeostasis) and behavioral traits (dietary intake, physical activity, sleep, and weight control behaviors) that influence energy homeostasis in 500 adults.

**Methods:**

A cohort of adult Samoans who participated in a genome-wide association study of adiposity in Samoa in 2010 was followed up, based on the presence or absence of the *CREBRF* variant, between August 2017 and March 2019. Over a period of 7-10 days, each participant completed the main study protocol, which consisted of anthropometric measurements (weight, height, circumferences, and skinfolds), body composition assessment (bioelectrical impedance and dual-energy x-ray absorptiometry), point-of-care glycated hemoglobin measurement, a fasting blood draw and oral glucose tolerance test, urine collection, blood pressure measurement, hand grip strength measurement, objective physical activity and sleep apnea monitoring, and questionnaire measures (eg, health interview, cigarette and alcohol use, food frequency questionnaire, socioeconomic position, stress, social support, food and water insecurity, sleep, body image, and dietary preferences). In January 2019, a subsample of the study participants (n=118) completed a buttock fat biopsy procedure to collect subcutaneous adipose tissue samples.

**Results:**

Enrollment of 519 participants was completed in March 2019. Data analyses are ongoing, with results expected in 2020 and 2021.

**Conclusions:**

While the genetic variant rs373863828, in CREBRF, has the largest known effect size of any identified common obesity gene, very little is currently understood about the mechanisms by which it confers increased odds of obesity but paradoxically lowered odds of type 2 diabetes. The results of this study will provide insights into how the gene functions on a whole-body level, which could provide novel targets to prevent or treat obesity, diabetes, and associated metabolic disorders. This study represents the human arm of a comprehensive and integrated approach involving humans as well as preclinical models that will provide novel insights into metabolic disease.

**International Registered Report Identifier (IRRID):**

RR1-10.2196/17329

## Introduction

### Background

As is now the case in many Pacific Island nations, the prevalence of obesity and diabetes in Samoa have reached epidemic proportions. In 2013 (the most recent estimates), 41.2% of men and 65.1% of women could be classified as having obesity based on Polynesian-specific body mass index (BMI) cutoff points (>32 kg/m^2^) [1]. Data from the same survey indicated that type 2 diabetes mellitus was present in 19.6% of men and 19.5% of women and projections suggest that the proportion of the population with diabetes may exceed 26% by 2020 [1].

The underlying etiology of the rapidly increasing prevalence of obesity and related cardiometabolic disorders in the Pacific generally [2,3], and Samoa specifically [1], can be attributed in large part to the rapidly changing economic and nutritional environment. While energy availability, often in the form of calorie-dense, nutrient-poor, imported foods has increased rapidly in recent decades [4], opportunities for subsistence agriculture-related physical activity have simultaneously declined, resulting in chronic positive energy balance and associated metabolic disease. Perhaps compounding the effect of the changing nutritional and physical activity environment, however, is a recently identified genetic variant, rs373863828 (CREB 3 regulatory factor, *CREBRF*: c.1370G>A p.[R457Q]), which is associated with increased odds of obesity, but paradoxically, decreased odds of diabetes [5].

The *CREBRF* missense variant (*CREBRF*), first identified in a genome-wide association study (GWAS) we conducted in 2010 [5], is common among Samoans (minor allele frequency=0.259) and has a larger effect on BMI than any previously identified common genetic variant. The minor allele (A) is associated with greater average adult BMI of 1.36 kg/m^2^ per copy, an effect size approximately 3-fold greater than variation near *FTO* (0.39 kg/m^2^ per copy) [6]. Interestingly, however, the higher-BMI allele is associated with *decreased* odds of type 2 diabetes and *lower* fasting serum glucose levels. Several studies in New Zealand Māori and Pacific Islanders (Tongans, Cook Islanders, and Niueans) have recently replicated these findings [7-11], demonstrating comparable minor allele frequencies and reproducing the incongruent effects on BMI and type 2 diabetes. The variant is nearly absent in non-Pacific Islanders (14 alleles in 198,121 individuals, allele frequency=0.00004 in BRAVO and the Genome Aggregation Database (gnomAD) combined) [12,13].

Findings from mouse cell models indicate that the *CREBRF* genetic variant plays a role in both adipogenesis and improving cell survival under starvation conditions [5]. It appears to function by reducing energy substrate utilization for basic cellular processes and reallocating energy to lipid storage, perhaps functioning as a “thrifty gene” [5]. How the gene functions on a whole-body level and the underlying explanation for its paradoxical association with obesity and diabetes, however, remains unknown.

### Objectives

The overarching goal of this study was to precisely characterize the impact of the *CREBRF* variant on metabolic (body composition and glucose homeostasis) and behavioral traits (dietary intake, physical activity, sleep, and weight control behaviors) that influence energy homeostasis in 500 adults. Specifically, we aimed to (1) determine if the *CREBRF* variant’s paradoxical association with BMI and diabetes could be explained by the amount (total fat mass) and distribution of adiposity (subcutaneous relative to visceral or central versus peripheral); (2) determine the association of the *CREBRF* variant with measures of glucose homeostasis and insulin action; (3) explore gene-environment interactions among the *CREBRF* variant and dietary intake, physical activity, and other behavioral traits; and (4) understand how the *CREBRF* variant influences metabolic homeostasis and gene expression in human adipose tissue.

## Methods

### Study Design

The Soifua Manuia (*Good Health* in Samoan) study described here follows a cohort of adult Samoans who participated in a GWAS of adiposity in Samoa in 2010 and who were followed up, based on the presence or absence of the *CREBRF* genetic variant, between August 2017 and March 2019. The recruitment procedures and protocols associated with the 2010 GWAS study have been described in detail previously [14]. *This study protocol describes the procedures used during the 2017-2019 interaction with study participants***.**

The study underwent initial and annual continuing ethical review by institutional review boards (IRBs) at Yale and Brown Universities (there was a reliance agreement between Brown and Yale, with Yale serving as the IRB of record, IRB #1604017547). Data analysis activities at the University of Pittsburgh were reviewed by their IRB and were determined to be exempt (IRB #PRO16040077) based on their receipt of only deidentified data. The PartnersHealth Office of Research approved protocols for sleep apnea studies, and all aspects of the protocol were reviewed and approved by the Health Research Committee of the Samoan Ministry of Health. All participants provided written informed consent for their participation.

### Setting

Samoa is an independent Pacific Island nation consisting of 2 large (*Upolu and Savai’i*) and several smaller islands. The majority of the Samoan population is rural (81%), and 78% are residents on the island of *Upolu* [15], which is divided into 3 census regions: the Apia urban area (AUA), Northwest Upolu (NWU), and the rest of Upolu (ROU).

Classified as an upper middle-income economy by the World Bank, Samoa’s gross national income was US $4120 per capita in 2017 [16], and the country ranked 104 out of 188 globally based on the Human Development Index (HDI value=0.704) [17]. In 2017, the population of Samoa was 195,352, with an anticipated annual growth rate of 0.9%. Life expectancy at birth in 2016 was 78 for women and 72 for men [18].

### Participants

Participants included in the 2017-2019 Soifua Manuia study were purposively recruited to study genetic and environmental influences on adiposity and cardiometabolic health. Specifically, we aimed to recruit a sample of 500 participants, with equal numbers of men and women, 100 of whom had 2 copies of the *CREBRF* minor/risk allele (AA), 200 of whom had one copy (AG), and 200 of whom had zero copies (GG). We carried out this nonrepresentative sampling by genotype to have higher power to detect contrasts between the genotype groups, while recognizing what was feasible in terms of recruitment given the overall rarity of the AA genotype in the population.

**Figure 1:**
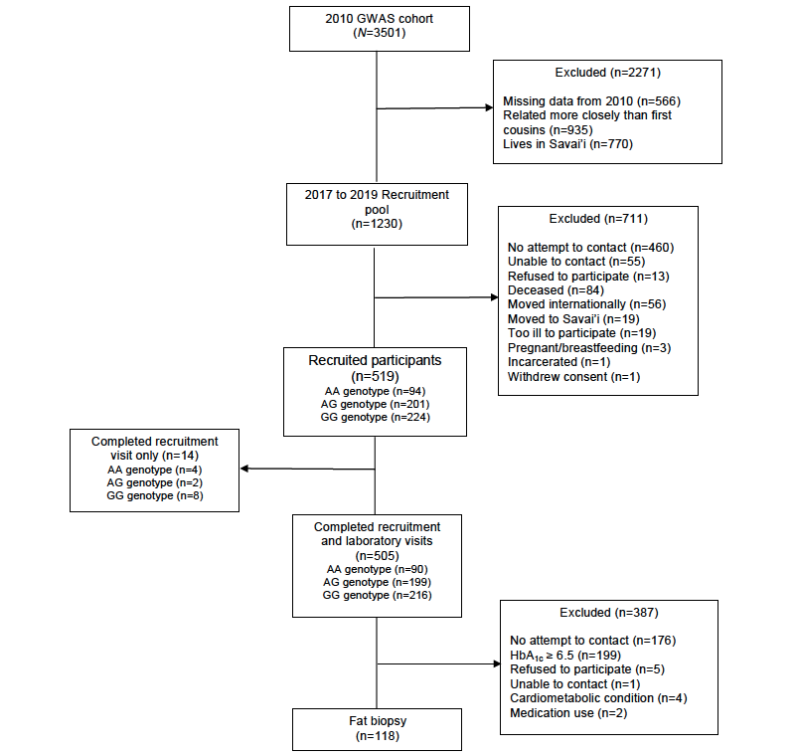
Consolidated Standards of Reporting Trials diagram describing recruitment into the Soifua Manuia study and completion of study procedures. GWAS: genome-wide association study; HbA^1c^: glycated hemoglobin.

*CREBRF* genotypes were determined based on participants’ data from the 2010 GWAS study [5,14]. The original eligibility criteria for the GWAS study included Samoan ethnicity (which was determined based on participant report that they had 4 Samoan grandparents), were aged 24.5 years to less than 65 years, nonpregnant, and had no physical or cognitive impairment that would prohibit completion of study procedures. To create a list of potential participants to be recruited for the 2017-2019 follow-up a number of selection criteria were applied to the 2010 data (Figure 1): only participants who consented in 2010 to being recontacted for additional research studies were included; participants must have had genotype data from the 2010 study and complete data on key outcomes of interest (BMI and serum glucose levels) and must have been resident on the island of Upolu (because study equipment could not be transported to the less developed and less densely populated island of Savai’i, where 9 of 33 of the 2010 GWAS villages were located). Then, kinship estimates were used (GenABEL [19]) to restrict the sample to those who were only minimally related to one another (less than first cousins, maximum kinship estimate 6.01%) because reducing the genetic correlation among the sampled individuals increases the effective population size. From this sample, individuals with the AA genotype were prioritized for recruitment. Early on in recruitment, we attempted to recruit 2 AG genotype and 2 GG genotype individuals for each recruited AA genotype individual, matching on sex, age within 5 years, and census region. Halfway through the recruitment, this protocol was adjusted to allow for faster recruitment, prioritizing AA and AG genotype individuals without explicitly matching. In this system, target recruitment lists were generated from the list of unrelated individuals and participants were recruited until the desired sample size for each genotype group within sex was achieved.

Recruitment occurred between August 2017 and March 2019 from 23 villages on the island of Upolu (8 urban [AUA], 7 periurban [NWU], and 8 rural [ROU]). After government and village-level permissions were granted, Samoan research assistants (2 out of 4 of whom had assisted with the 2010 GWAS study) used phone calls and visits to the participants’ 2010 villages of residence to relocate participants. Village leadership (village mayors and women’s committee members) assisted research staff in locating participants’ homes. If a participant could not be located, the reason was noted (Figure 1). In the case of participant death, a death certificate was requested from the Samoa Bureau of Statistics to document the cause.

During visits to participants’ homes, research staff provided detailed information about the purpose of the 2017-2019 follow-up study and its protocols, gained written informed consent from all participants (including permission to link their 2017-2019 phenotype data with their 2010 data, which had been shared with the database of Genotypes and Phenotypes (dbGap) [20]), and screened the participants for eligibility. The same eligibility criteria were applied in 2017-2019 as in 2010 [14]; pregnant women were excluded, as were those who had developed physical or cognitive impairments preventing full participation in study procedures. Additional exclusion criteria included having given birth in the past 6 months or current lactation, ongoing use of weight loss medications, weight loss surgery, or significant recent weight loss (>5% of body weight in the past year). Seven to nine years after their original participation in the 2010 GWAS study, participants’ age ranged from 30.7 years to 72.7 years. A total of 519 participants agreed to participate in the study (Figure 1): 94 participants with the AA *CREBRF* genotype, 201 with the AG genotype, and 224 with the GG genotype.

### Study Procedures

Willing and eligible participants completed study activities over the course of 7-10 days. Two in-person visits took place, one in the participant’s home and the other 7-10 days later in the Obesity, Lifestyle, and Genetic Adaptations (OLaGA; *n*. *life* in Samoan) research laboratory, located in the capital city of Apia, and research assistants contacted participants once by phone between the in-person visits. During the home visit, research assistants collected height, weight, hemoglobin (for assessment of anemia), administered a number of questionnaires, and initiated an accelerometer-based device, which participants wore to objectively measure physical activity for at least seven days before their visit to the OLaGA laboratory. Remaining study procedures were completed during the laboratory visit. A subsample of participants provided a subcutaneous buttock fat biopsy, which was collected at a separate research visit in January 2019. Participants were compensated for time spent completing the protocol, receiving 75 Western Samoan Tala (WST; approximately US $28) for their participation as well as a 10 WST phone card to allow them to complete dietary recalls by telephone. Table 1 provides an overview of the study activities and their timing, and each of the procedures are described in detail below.

**Table 1:** Summary of the Soifua Manuia study procedures.

Measures		2010		Home visit, 2017-2019		Laboratory visit, 2017-2019		Fat biopsy visit, 2019	
**Anthropometric measures**									
	Weight		X^a^		X		X		X
	Height		X		X		X		X
	Circumferences (midupper arm, abdominal, hip, and midcalf)		X		—^b^		X		—
	Skinfold thicknesses (forearm, tricep, subscapular, abdominal, and suprailiac)		X		—		X		—
**Body composition**									
	BIA^c^		X		—		X		—
	DXA^d^ (total body, lumbar spine, hip, and forearm)		—		—		X		—
**Blood pressure and hand grip strength**									
	Blood pressure and heart rate		X		—		X		—
	Hand grip strength		—		—		X		—
**Biospecimen collection**									
	Hemoglobin		—		X		—		—
	Point-of-care HbA_1c_^e^		—		—		X		X
	Point-of-care fasting blood glucose		X		—		X		—
	Serum		X		—		X		—
	OGTT^f^		—		—		X		—
	DNA		X		—		X		—
	RNA		—		—		X		—
	Urine		—		—		X		—
	Objective physical activity monitoring (ActiGraph GT3X+)		—		X		X		—
	Sleep monitoring (WatchPAT)		—		—		X		—
	24-Hour dietary recalls		—		X		X		—
**Questionnaire measures^g^**									
	Demographic information		X		X		—		—
	Health Interview^h^		X		X		—		X
	Women’s health		X		X		—		—
	Cigarette and alcohol use		X		X		—		—
	Physical activity		X		X		—		—
	Food frequency questionnaire		X		X		—		—
	Material lifestyle		X		X		—		—
	Health care choices		—		—		X		—
	Stress		—		—		X		—
	Social support and conflict		—		—		X		—
	Health locus of control		—		—		X		—
	Self-efficacy		—		—		X		—
	Food and water insecurity		—		—		X		—
	Sleep		—		—		X		—
	Body image		—		—		X		—
	Weight stigma and attitudes		—		—		X		—
	Weight control behaviors		—		—		X		—
	Dietary preferences		—		—		X		—
	Living with diabetes		—		—		X		—
	Bone and muscle function		—		—		X		—
	Buttock fat biopsy		—		—		—		X

^a^Indicates that the measure was completed.

^b^Indicates that the measure was not collected at the specific visit.

^c^BIA: bioelectrical impedance analysis.

^d^DXA: dual-energy x-ray absorptiometry.

^e^HbA_1c_: glycated hemoglobin.

^f^OGTT: oral glucose tolerance test.

^g^Questionnaire measures are described in detail in Table 2.

^h^An abbreviated health history was collected for the purposes of additional eligibility screening before the buttock fat biopsy.

### Body Size and Body Composition

Protocols used in 2010 for the collection of anthropometric data were replicated during the 2017-2019 follow-up study [14]. Weight and height were measured in duplicate to the nearest 0.1 kg and 0.1 cm, respectively, using a digital weighing scale (Tanita HD 351; Tanita Corporation of America) and a portable stadiometer (SECA 213, Seca GmbH & Co). Circumferences (midupper arm, abdomen [at the level of the umbilicus], hip, and midcalf) and skinfold thicknesses (forearm, tricep, subscapular, abdominal, and suprailiac; Lange calipers, Beta Technology Inc) were also measured in duplicate to the nearest 0.1 mm. Measurements exceeding the maximum capacity of the skinfold calipers (>65 mm) were recorded as doing so in participant records. Bioelectrical impedance analysis (BIA) measures of resistance and reactance were obtained with an RJL BIA-101Q device (RJL Systems) in all participants without metal implants or pacemakers, using standard procedures. These data will be used to estimate fat mass and body fat percentage.

Whole body and regional body composition were also assessed using dual-energy x-ray absorptiometry (DXA; Lunar iDXA, Encore version 17, General Electric (GE) Healthcare Medicine). Additional eligibility criteria were applied to this assessment; participants did not undergo a DXA scan if they had been exposed to additional x-ray or computed tomography scans in the past 12 months (with the exception of dental x-rays). All women of childbearing age (<55 years) were tested for pregnancy before completing the DXA scan. Participants wore standardized clothing, without metal inserts or buttons, for a total body scan administered by 1 of 4 trained DXA operators in *standard* or *thick* mode. Body composition outcomes of interest were fat mass, lean mass, and visceral fat mass and volume (estimated using the CoreScan application, GE Healthcare Medicine). In cases where the participant’s body size exceeded the limits of the scan area, participants were placed on the scanning bed such that the first scan captured the limbs on one side of the body and as much of the trunk region as possible. Participants were then repositioned to scan the remaining half of the body. Analyses in these cases will proceed using a mirror mode, where total body composition will be estimated from one half of the body. Among obese adults in other settings, this technique results in nonsignificant differences between mirrored and standard scan data [21].

DXA was also used to assess the bone mineral density and bone mineral content of the whole body, lumbar spine, nondominant hip, and forearm. Bone geometry at the femoral neck was estimated using Hip Structural Software (GE Healthcare Medicine) to calculate section modulus (bone resistance to bending), cross-sectional area, cross-sectional moment of inertia, and minimal neck width.

### Blood Pressure and Hand Grip Strength

After a 10-min seated rest period, blood pressure and heart rate were measured 3 times, with a 3-min rest period between measurements, using an Omron HEM-907XL automated blood pressure monitor (Omron Healthcare). Hand grip strength was assessed as a functional measure of overall strength. After adjusting the handle position of a Jamar Plus+ digital hand dynamometer (Patterson Medical) according to participants’ optimal comfort, maximal grip strength was measured in triplicate for both hands with 30-second rest periods between measurements. Procedures recommended by the American Society of Hand Therapists were followed for standardization [22].

### Biospecimen Collection

Point-of-care testing devices requiring fingerstick blood samples were used to measure hemoglobin (for assessment of anemia; AimStrip Hb test system, Germaine Laboratories Inc) and glycated hemoglobin (HbA_1c_; DCA vantage analyzer, Siemens Healthcare GmbH). HbA_1c_ values were used to determine if an oral glucose tolerance test (OGTT) could be administered safely: if HbA_1c_ was ≥9.0%, participants did not complete the OGTT, and if HbA_1c_ was ≥6.5% (indicating diabetes) but <9.0%, fasting blood glucose (FBG) was also measured (Bayer Contour Next, Bayer HealthCare LLC) and only participants with FBG <200 mg/dL were allowed to complete the OGTT.

All participants completed a fasting blood draw, where Samoan phlebotomists collected whole blood for serum separation, DNA extraction, and RNA expression (using PaxGene (R) Blood RNA vacutainers, PreAnalytiX GmbH). Participants who could safely complete the 2-hour 75 g OGTT followed protocols recommended for diabetes diagnosis by the American Diabetes Association [23]. Additional whole blood was collected at 30-min intervals throughout the 2-hour protocol (30-, 60-, 90-, and 120-min postglucose load). Serum from the fasting samples and OGTT draws was separated by centrifugation and stored at −80°C before transport on dry ice to the University of Pittsburgh for analysis. Fasting samples are currently being analyzed for glucose, insulin, free fatty acids, lipid levels (total-, high density lipoprotein, low density lipoprotein, and very low density lipoprotein cholesterol, triglycerides), adiponectin, leptin, and markers of liver function (alanine amino transferase and aspartate aminotransferase). Glucose, insulin, and free fatty acids are being measured in all postglucose load samples.

Whole blood samples for DNA extraction were processed to the cell lysate stage using red blood cell lysis, protein precipitation, and cell lysis solutions following manufacturers protocols (5Prime T-MArchivePure TM, Thermo Fisher Scientific) and shipped at room temperature to the University of Cincinnati where further extraction steps were completed. PAXgene (R) vacutainers were chosen to collect whole blood samples for RNA expression because of their stability during transportation and storage. However, after the initiation of this protocol we learned of United States’ import restrictions on the vacutainer reagents, meaning that processing had to be completed at the Samoan site. After storage at −80°C, samples were thawed and incubated at room temperature for up to 24 hours. RNA was isolated using the PAXgene (R) blood miRNA kit (PreAnalytix) according to the manufacturer’s protocol with the following modification: step 5 of the Qiagen protocol stipulates, “Pipet the sample into a 1.5 ml microcentrifuge tube. Add 300 μl Buffer BM2 and 40 μl proteinase K. Mix by vortexing for 5 s, and incubate for 10 min at 55°C in a shaker incubator at 400-1400 rpm.” A shaker incubator was unavailable for this step; so, after the addition of proteinase K, samples were incubated for 5 min at 55°C, uncapped, recapped, vortexed, and incubated for another 5 min at 55°C. Two elutions of 40 µl of buffer BR5 produced a final volume of 80 µL RNA. Following isolation, RNA was stored at −80°C before transfer to a GenTegra RNA matrix. RNA was applied to the GenTegra matrices per the manufacturer’s protocol, distributing each sample among 2 GenTegra tubes (approximately 40 µl each), and dried overnight in a biosafety cabinet by evaporation. Tubes were then capped, stored, and inventoried before transport to the University of Pittsburgh. Recovery of RNA was performed according to the manufacturer’s instructions, using the same volume buffer BR5 as was originally added to the matrix.

Random urine specimens were collected and stored for the study biobank. To allow for the later examination of exposure to environmental chemicals, samples were collected in polypropylene containers, and participants were asked not to touch the inside of the specimen cups or cup lids. Urine specific gravity was measured using a handheld digital refractometer (ATAGO, PAS-10S, ATAGO Co. Ltd) to quantify urine dilution. Samples were aliquoted into polypropylene cryovials and stored at −80°C before shipping on dry ice to Brown University.

### Questionnaires

Over the course of the 2 study visits and in the midparticipation phone call, participants responded to questionnaires that documented their individual and household demographic and socioeconomic characteristics, current health status and health history, physical activity, dietary intake, and sleep patterns and symptoms. Participants also completed multiple psychosocial measures, including body image, stress, self-efficacy, and weight control behaviors. All questionnaire measures were chosen based on existing literature linking the concepts measured with obesity, diabetes, or other aspects of cardiometabolic health and well-being. All the questionnaire measures employed are listed in Table 2, with a brief summary of their content and references for the original survey instruments.

All questionnaires were translated into the Samoan language and administered in Samoan by bilingual local research assistants. Data were collected on iPads using Research Electronic Data Capture (REDCap) tools hosted by Yale University [24,25]. Each questionnaire was completed once, either at the home visit or laboratory assessment, with the exception of the 24-hour dietary recalls, which were completed on three occasions—twice in person (at the home and laboratory visits) and once by phone on a randomly selected day between the 2 in-person visits. Recall days were selected to include 2 weekdays and one weekend day, and protocols followed the multiple-pass method recommended by the US Department of Agriculture [26]. The timing of administration of each questionnaire was carefully considered. First, questionnaires that were asked in 2010 were repeated at the first contact with participants to protect against any loss to follow-up that may have occurred between the home and laboratory visits and maximize the longitudinal data available for assessment (weight and height were collected at the first contact for a similar reason). Second, questionnaires that may have triggered alterations in behavior during the period of measurement between visits (such as some of the weight control behavior or body image measures) were only asked at the laboratory visit once monitoring of dietary intake and physical activity were complete.

**Table 2:** Soifua Manuia study questionnaire measures completed in 2017-2019.

Questionnaire	Number of items	Asked in 2010	Description	References^a^
Demographic information	6	X^b^	Basic demographic information including age, sex, marital status, and education	—^c^
Health interview^d^	36	X	Diagnosis with medical conditions (diabetes, hypertension, heart disease, and high cholesterol), medication use, use of traditional healers, and self-reported health and weight	—
Women’s health^d^	26	X	Parity, gravidity, age at menarche, menstrual regularity, contraceptive use, infertility, pregnancy history, and menopause (age and symptoms)	Barkan et al [27]
Cigarette and alcohol use	15	X	Use of tobacco products, alcohol consumption, and problems with drunkenness	—
Physical activity	11	X	Global Physical Activity Questionnaire (GPAQ), which estimates time spent in moderate-vigorous work, transport, and leisure-related activities	World Health Orgnaization [28]
Food frequency questionnaire	111	X	Locally validated food frequency questionnaire measuring consumption (never or less than once a month to more than 6 times per day) and usual portion size	—
Material lifestyle^d^	46	X	Household assets inventory, amenities (plumbing, cooking, and toilet facilities), number of household residents, occupation of heads of household, income, village wealth, disparities, and community spirit	—
Health care choices	17	—	Use of and preferences for traditional versus western biomedical health care practitioners	—
Stress	18	—	Cohen Perceived Stress Scale (PSS) and Short Form-8 quality of life measures	Cohen et al [29]; Ware et al [30]
Social support and conflict	19	—	Multidimensional Scale of Perceived Social Support (MSPSS) and perceived social conflict	Zimet et al [31]; O'Brien et al [32]
Health locus of control	18	—	Beliefs about internal versus external control over health and well-being	Wallston et al [33]
Self-efficacy	35	—	General and social self-efficacy and exercise confidence survey	Sherer et al [34] Clark et al [35]; Sallis et al [36]
Food and water insecurity	52	—	Household Food Insecurity Access Scale (HFIAS) and Household Water Insecurity Experiences scale (HWISE)	Coates et al [37]; Young et al [38]
Sleep	23	—	Sleep duration, sleep quality, chronotype, shift work, and Epworth Daytime Sleepiness Scale (EDSS)	Bild et al [39]; Johns [40]
Body image	20	—	Satisfaction with body shape and size and body size preferences	Brewis et al[41]
Weight stigma and attitudes^e^	66	—	Attitudes toward obese persons scale, beliefs about obese people, weight stigma, and discrimination	Allison et al [42]; Myers and Rosen [43]
Self-esteem	19	—	Rosenberg self-esteem scale	Rosenberg [44]
Weight control behaviors	53	—	Eating habits, dietary preferences, and weight control efforts	Neumark-Sztainer et al [45]
Dietary preferences	50	—	Three-factor eating questionnaire (cognitive restraint of eating, disinhibition, and hunger)	Stunkard and Messick [46]
Living with diabetes^f^	86	—	Diabetes symptoms and self-care behaviors, perceived diabetes control, medication adherence, and beliefs about diabetes	Carey et al [47]; DePue et al [48]
Bone and muscle function	9	—	Broken bones, bone-specific supplement use, and SARC-F screen for sarcopenia	Malmstrom et al [49]

^a^References indicate the sources of questionnaire items; questionnaires may not have been used in their entirety and may have been adapted to the Samoan context.

^b^Indicates that the questionnaire was also completed in 2010.

^c^Indicates that the measure was not collected in 2010. In the references column, it indicates that questions cannot be attributed to a single source or were of the authors design.

^d^Some questionnaire items were updated between 2010 and 2017, so not all questions were consistent across time points; health interviews were updated with additional questions about medication adherence and locally available generic medication brands, women’s health included more detailed menstrual regularity questions in 2010, and the material lifestyle household assets inventory was updated with newly available household commodities monitored in the Samoa Demographic and Health Survey.

^e^One section of this questionnaire (n=19 questions), concerned with experiences of overweight or obesity, was only asked of participants who self-reported being moderately or much too heavy.

^f^Asked only of those participants who self-reported a diagnosis of type 2 diabetes.

### Objective Physical Activity Monitoring

Actigraph GT3X+ accelerometer-based devices (ActiGraph Corporation) were used to objectively measure physical activity during the 7-10 day period between the home and laboratory visits. The GT3X+ was secured to the nondominant wrist using a disposable hospital band. The wrist placement was chosen for several reasons: (1) for continuous monitoring that would allow assessment of sleep patterns, (2) to better accommodate Samoan clothing styles (as compared with waist-worn), and (3) to prevent loss of the devices and minimize missing data associated with participants forgetting to replace their device after removing with clothing or bathing. The GT3X+ devices were initialized to collect raw acceleration at 30 Hz, and participants were asked to wear the devices continuously until the laboratory visit. Devices were removed at the laboratory visit, where data were downloaded and briefly reviewed for completeness. In the event that less than 5 days of data were recorded, participants were asked to repeat the assessment. Expected output from the objective physical activity monitoring includes estimates of daily energy expenditure (metabolic equivalents); daily steps; sedentary time; and time spent in light, moderate, and vigorous physical activity.

### Sleep Apnea Detection

We provided 391 participants with a WatchPAT TM 200 Unified (Itamar Medical Ltd.) device, consisting of a finger probe and chest sensor to evaluate sleep apnea and sleep patterns (devices became available several months into the recruitment period, after which all participants completed this assessment). The WatchPAT TM was worn on the nondominant wrist (proximal to the GT3X+, so results could be compared) the night before the laboratory visit. The device measured peripheral arterial tone (PAT TM) signal, oxygen saturation, actigraphy, acoustic decibels (snoring evaluation), and body position. The output was downloaded to a computer, encrypted, and sent to collaborators at Brigham and Women’s/Harvard Medical School for interpretation. Sleep studies were reviewed within 48 hours of receipt by expert analysts, and all participants with readings indicative of severe sleep apnea (n=15) were referred to a local sleep clinic in Apia.

### Buttock Fat Biopsies

At the time of consent for the overall study, participants were asked to consent separately to completing a subcutaneous buttock fat biopsy so that the effect of the *CREBRF* variant on gene expression in adipose tissue could be determined. To limit variation by disease status, we selected a subsample of participants to recontact and ask to participate in this additional procedure. The participants in this subset were those without diabetes (those without a self-reported diabetes diagnosis at the time of participation in the main protocol and those with HbA_1c_ <6.5%), cancer, or cardiovascular disease. For convenience and efficiency, we sampled participants within a 45-min drive of the AUA. Participants were recontacted either by phone or at their homes during a 2-week period in January 2019. Given the genotype frequencies and diabetes prevalence in the population, we aimed to recruit 26 nondiabetic women and 15 nondiabetic men for each of the 3 genotypes (AA, AG, and GG). Participants were rescreened for the overall study eligibility criteria (pregnancy, lactation, birth within 6 months, and major weight loss) as well as the specific biopsy criteria, including additional point-of-care HbA_1c_ measurement, in their homes. Willing and eligible participants were then scheduled for the biopsy procedure.

Biopsies were completed in a sterile environment at a local medical facility. After the procedure, an adapted version of that described by Beynen and Katan [50] was explained in detail by the study clinician (A Prescott, plastic surgery resident), and additional verbal consent to the procedure was obtained in the Samoan language by study staff. Participants lay in a prone position with the left gluteal soft tissue exposed. Using red light technology, a commercial vein identification device (Illumivein, Easy-RN LLC) was used to identify any venous plexuses in the subcutaneous tissues. The anticipated biopsy site (with the least amount of subcutaneous vessels) was marked with a surgical skin marker, and an ice pack was applied for 1 min to numb the area. The skin was prepped with single-use sterile ChloraPrep (TM; Becton, Dickinson and Company), allowed to dry, and topical Gebauer’s ethyl chloride spray was used to anesthetize the anticipated biopsy site. Participants were asked to contract their gluteal muscles, while the clinician performed a pinch test (using single-use, latex-free, sterile gloves) on the anticipated biopsy site. A single-use, sterile 16-gauge needle attached to a single-use, sterile 1 cc syringe was inserted into the biopsy site percutaneously to harvest the adipose tissue samples. Using a handheld aspiration technique (applying gentle negative manual pressure to the syringe or needle apparatus), 10-30 passes were performed at a 45-degree angle, until adipose tissue was visibly seen in the syringe. In cases where the study participant demonstrated more than minimal discomfort (or if samples were too sanguineous), the biopsy was terminated immediately. Once the lipoaspirate was visible in the syringe, the needle or syringe was completely removed from the biopsy site and handed off to a technician for downstream processing. When the biopsy site was deemed adequately hemostatic, a small gauze was applied and secured gently with adhesive tape. Study participants were observed for up to 60 min before departing the study site. Study participants were provided with postbiopsy care instructions and a contact number to call if any unusual or severe adverse effects were encountered.

Following sample collection, the syringe containing the adipose sample was washed with 0.5-1 ml RNALater (R; Ambion Inc). The mixture of RNALater (R) and lipoaspirate was then expelled into a 1.5 5-ml microcentrifuge tube and incubated at 5°C overnight before transfer to −80°C the next day per the manufacturer’s protocol. Samples in RNALater (R) were transported from Apia, Samoa, to the University of Pittsburgh. Although samples in RNALater (R) are stable for up to a week at room temperature, we shipped the samples on ice, with refrigeration and/or refreshed ice between destinations, to maintain a more consistent temperature as temperatures can fluctuate dramatically across climates and altitudes. Upon arrival in Pittsburgh, samples were stored at −80°^o^C until processing. To process, frozen samples were thawed at room temperature, and RNALater (R) was removed from the sample by straining the RNALater (R) + lipoaspirate through a 0.22- µm nylon cell strainer placed atop a 50- ml conical tube (such that the sample was collected in the strainer and the RNALater was collected in the conical tube). The strainer was weighed before and after the sample was strained to acquire the mass of the lipoaspirate. The tissue was then collected from the strainer into a 14-ml round bottom tube containing 1 ml of QIAzol Lysis Reagent (Qiagen Sciences Inc) and homogenized for 30 seconds with a TissueRuptor (R; Qiagen Sciences Inc). Following homogenization, total RNA was isolated from the homogenate using RNeasy Lipid Tissue Mini Kit (R; also Qiagen) according to the manufacturer’s protocol, including optional DNase treatment to remove contaminating genomic DNA and optional full speed spin to remove residual buffer RPE or flow-through. Two elutions of 30-µl nuclease-free water were used in the final step for a final volume of 60 µl RNA.

### Participant Feedback and Referrals to Clinical Services

Participant feedback occurred in several stages. First, at the completion of the laboratory visit, all participants received a feedback sheet that used a *traffic light* (green, orange, and red) approach to describe their risk of overweight or obesity, anemia, hypertension, and diabetes (based on HbA_1c_) as well as recommendations for further follow-up screening and behavioral modifications based on those risks. After serum analyses were completed, participants received individual letters with their cholesterol, triglycerides, fasting glucose, and insulin, and OGTT results as well as a study summary, which described key findings from the overall study and provided village and like-for-like comparisons to allow participants to put their individual results in context (eg, men <40 years of age were provided with summary data for all men in their comparable age range). At both stages, study staff provided referrals to local clinical services where participants met local risk criteria for diabetes, hypertension, or anemia. Participants are able to engage with study progress via the study’s Facebook page (@YaleOlaga), and the private message function on the page offered an additional way for participants to contact study staff with questions about their participation (study staff could also be contacted by phone or text message).

### Data Management and Statistical Analysis

Descriptive analyses will first be used to characterize the metabolic and behavioral traits of participants. Questionnaire measures that have not previously been validated in the Samoan setting will be examined for validity and internal consistency before analyses proceed. Then, we will test for cross-sectional associations of measured traits with the *CREBRF* variant using methods appropriate for the categorical or continuous nature of the outcomes, while adjusting for relevant covariates including age, sex, and relatedness. While many of our analyses using the newly collected data will initially be hypothesis-free, we will continue to be informed by our parallel mouse and cell models and test the resulting hypotheses using methods appropriate to the questions being addressed. Longitudinal analyses, where there are comparable measures available from the 2010 data collection (eg, weight or BMI) will use appropriate linear-mixed models that explicitly model the dependence between the different time points. After quality control and data cleaning are completed, the cleaned datasets generated during this study will be available in the dbGaP repository under the accession number phs000914 [20].

## Results

The study was funded by the US National Institutes of Health (R01HL093093) in March 2016. Data collection was completed among 519 participants between July 2017 and March 2019, and results addressing the main study hypotheses are expected to be published in 2020 and 2021.

## Discussion

### Significance

Although the genetic variant rs373863828, in *CREBRF*, has the largest known effect size of any identified *obesity gene*, very little is currently understood about the mechanisms by which it confers increased odds of obesity but paradoxically lowered odds of type 2 diabetes. A small number of studies have evaluated the *CREBRF* gene (not this variant specifically) in nonhuman preclinical models. These studies have implicated *CREBRF* in a variety of disparate cellular and physiological processes, including the unfolded protein response [51], glucocorticoid signaling and maternal behaviors [52], reproduction [53,54], viral pathogenesis [55], angiogenesis [56], carcinogenesis [57,58], and starvation resistance [59]. However, this protocol is the first attempt to broadly characterize the association of the variant with a wide variety of metabolic and behavioral traits in humans.

Overweight, obesity, and type 2 diabetes—the complex and chronic conditions that are the focus of this protocol—are major health concerns that are estimated to cause 3.4 million deaths, 4% of years of life lost, and 4% of disability-adjusted life years in high-, low-, and middle-income countries [60]. Every effort must be made to understand the basic biological underpinnings of these complex, chronic conditions, so that appropriate pathways may be targeted by behavioral and pharmacologic intervention. Although we do not describe our additional ongoing studies here, the activities described in this protocol occurred alongside additional cell and mouse studies, including characterization of the gene networks mediating the effects of the variant on energy and metabolic homeostasis. We will also seek to identify the role of natural selection in the emergence of the *CREBRF* variant and other variants coselected with it that may contribute to its risk.

### Strengths and Limitations

Although we believe the approach we have described has the potential to generate new understanding of the genetic basis of obesity and diabetes risk in this population, it is not without limitations. Although several key analyses will be able to take advantage of longitudinal data, many of the phenotypes collected here were measured for the first time in the cohort in 2017-2019, meaning analyses will be cross-sectional and we will have limited ability to infer causality. Several of our measurement tools, particularly those used to measure behavioral traits, were new to the Samoan setting, and despite rigorous protocols for translation and staff training, will require testing for validity and reliability in this population. Most significant though are potential biases in our sampling, stemming from both our initial sampling strategy in the 2010 GWAS study, where a nonrepresentative convenience sampling approach was used, and our selective follow-up of only Upolu residents (necessitated by the near-impossibility of transporting the DXA scanner to the most rural residents of Savai’i). Our nonrepresentational sampling, with strong oversampling of AAs and moderate oversampling of AGs, ensures that we have enough individuals with the rarer genotypes to be able to contrast them against the common GGs. However, it is important to consider the possible effects of the initial matching on sex, age within 5 years, and census region, followed by looser matching; this sampling strategy, which may induce bias, reflects the realities and complexities of fieldwork. Kuo et al [61] found that *matching in loose-matching data can be ignored for negligible loss in testing and estimation if the distributions of matching variables are not extremely different between cases and controls*. To examine this in our own data, we simulated 20,000 replicates of a quantitative trait in the whole sample gathered in 2010 as a function of each individual’s observed values for age, sex, rs373863828 genotype (coded 0,1,2), census region, and random normal N(0,1) noise and assumed *true* effect sizes; the effect size of the genotype was set such that its mean *P* value was 2.7 × 10^−6^ in the larger 2010 sample. Then, we subset the data to the 519 individuals who are in our current sample and examined the percent bias in the estimates of the regression parameters derived ignoring matching. Similar to Kuo et al’s results [61], we find that the bias in the estimates is very small, for example, for the genotype effect size estimate, which is of primary interest, the bias is only −0.43%. Although more extensive simulations are merited, it appears that our sampling strategy does not induce strong bias in the estimates relative to their true population-level values. The estimated power in the 519 selected individuals to detect association of the simulated trait with genotype, while adjusting for the other predictors, at the .01 level was 82.8%. This is in line with earlier simulations done when we initially sought funding for this work where we found that, based on simulated sampling from the total set of the 2010 participants, we should have 87.1% power at the 0.01 level to detect our BMI signal and 78.4% power for hip circumference.

This study lays the groundwork for subsequent analysis to understand how the *CREBRF* risk variant influences adiposity and cardiometabolic traits. These studies, in combination with studies to understand the *CREBRF* gene more generally, are expected to be significant because they are likely to reveal entirely new pathways upstream and downstream of *CREBRF* that more broadly influence energy and metabolic traits across multiple populations and ethnic groups and individuals. Such knowledge could provide novel targets to prevent or treat obesity, diabetes, and associated metabolic disorders. This study represents the human arm of a comprehensive and integrated approach involving humans as well as preclinical models that will provide novel insights into metabolic disease.

## Abbreviations

AUA: Apia urban area
BIA: bioelectrical impedance analysis
BMI: body mass index
CREBRF: Creb 3 Regulatory Factor
DXA: dual-energy x-ray absorptiometry
FBG: fasting blood glucose
GWAS: genome-wide association study
HbA1c: glycated hemoglobin
IRB: institutional review board
NWU: Northwest Upolu
OGTT: oral glucose tolerance test
OLaGA: obesity, lifestyle, and genetic adaptations
ROU: rest of Upolu
WST: Western Samoan tala
